# The 3D Structure of the Apical Complex and Association with the Flagellar Apparatus Revealed by Serial TEM Tomography in *Psammosa pacifica*, a Distant Relative of the Apicomplexa

**DOI:** 10.1371/journal.pone.0084653

**Published:** 2014-01-02

**Authors:** Noriko Okamoto, Patrick J. Keeling

**Affiliations:** The Department of Botany, University of British Columbia, Vancouver, British Columbia, Canada; University of Melbourne, Australia

## Abstract

The apical complex is one of the defining features of apicomplexan parasites, including the malaria parasite *Plasmodium*, where it mediates host penetration and invasion. The apical complex is also known in a few related lineages, including several non-parasitic heterotrophs, where it mediates feeding behaviour. The origin of the apical complex is unclear, and one reason for this is that in apicomplexans it exists in only part of the life cycle, and never simultaneously with other major cytoskeletal structures like flagella and basal bodies. Here, we used conventional TEM and serial TEM tomography to reconstruct the three dimensional structure of the apical complex in *Psammosa pacifica*, a predatory relative of apicomplexans and dinoflagellates that retains the archetype apical complex and the flagellar apparatus simultaneously. The *P. pacifica* apical complex is associated with the gullet and consists of the pseudoconoid, micronemes, and electron dense vesicles. The pseudoconoid is a convex sheet consisting of eight short microtubules, plus a band made up of microtubules that originate from the flagellar apparatus. The flagellar apparatus consists of three microtubular roots. One of the microtubular roots attached to the posterior basal body is connected to bypassing microtubular strands, which are themselves connected to the extension of the pseudoconoid. These complex connections where the apical complex is an extension of the flagellar apparatus, reflect the ancestral state of both, dating back to the common ancestor of apicaomplexans and dinoflagellates.

## Introduction

The apicomplexans are a group of obligate intracellular parasitic protists that includes human parasites such as malaria parasites *Plasmodium*, a major disease for which about half the world population is at risk [1]. Other major human pathogens include *Toxoplasma gondii*, which infects up to a third of world population [2], and *Cryptosporidium*, a major contaminant of drinking water. Apicomplexans also include important veterinary parasites, such as *Eimeria* (estimated to cause more than US $3 billion annual loss worldwide to modern poultry industry [3]), *Cryptosporidium*, *Babesia*, and *Theileria*, all of which cause economic loss in variety of livestock.

Apicomplexan parasites are characterized by a subcellular structure called the apical complex. The apical complex was first observed in thin sectioned *Toxoplasma gondii* under transmission electron microscopy (TEM) [4] and intensively studied in other apicomplexan parasites from the late 1950’s through the 1970’s (for review, [5]–[7]). The apical complex of *T. gondii* is composed of the conoid, which is a closed, truncated cone composed of the unique fibres of tubulin polymers [8] with terminal rings on the anterior apex; rhoptries, which are electron dense, rhomboid-shaped vesicles with a narrow anterior neck and a wider posterior end; micronemes, which are also electron dense vesicles; and dense granules, which are spherical vesicles larger than micronemes and containing electron dense materials [9]. There is also diversity among the Apicomplexa, e.g., *Plasmodium* lacks the conoid or *Theileria* lacks micronemes. The apical complex is fundamental to apicomplexan infection, because it mediates the processes of host attachment and invasion (for recent reviews see [10], [11]). Accordingly, the molecular components of the apical complex and their role in the invasion machinery have been intensively investigated based upon cell biological, genomic, transcriptomic, and proteomic information accumulated in the last decade [12], [13].

From an evolutionary perspective, the origin of the apical complex and its relation to other cytoskeletal components, especially the flagellar apparatus are of great interest. The flagellar apparatus is a fundamental part of the eukaryotic cytoskeleton. It is located at the base of the flagella and typically composed of the basal body, microtubular roots, and fibrous connective structures. Moestrup [14] established a universal numbering system for the microtubular roots based on the generation of the basal bodies. This system enables us to use the flagellar apparatus to infer the evolution of the system, and is accepted as a standard for describing the flagellar apparatus across diverse eukaryotes [15]. Functionally, the flagellar apparatus involves multiple roles such as flagellar movement, feeding behavior, and microtubule organizing centers (MTOC) during cytokinesis. At the same time, the flagellar apparatus provides the common ground to understand the homology of cell architecture in distant relatives, especially cytoskeletal elements [15].

Recently, some components of the flagellar apparatus, i.e., striated fibre assemblins [16] and SAS6-like [17] were demonstrated to localize to the apical complex, which strongly suggests that the apical complex evolved from the flagellar apparatus. However, no apicomplexan is known to have the apical complex and the flagellar apparatus at the same time: the apical complex exists only in the invasive stages where the flagellar apparatus is morphologically reduced to a pair of centrioles, and flagella are only known in some gametes that lack an apical complex. So this hypothesis is difficult to test directly in the apicomplexans. But the apical complex is not restricted to apicomplexans, and is also found in a small and little studied collection of free-living relatives. Apicomplexans are members of a larger group, the alveolates, which also includes ciliates and dinoflagellates, within which apicomplexans and dinoflagellates are sisters and form a group with a handful of lesser-known organisms, collectively called myzozoans [18]. Myzozoans are characterized by “myzocytosis”, a mode of predation originally described in dinoflagellates [19], where the predator pokes a hole in the plasma membrane of a prey cell to suck out its cytoplasm into a food vacuole. These predators have been found to use a variant of the apical complex to mediate myzocytosis [18], [20]–[24]: apical complex-mediated host invasion, it turns out, is a variation of myzocytosis in the opposite direction.

The “archetype” apical complex in these lineages consists of an open-sided conoid, or pseudoconoid [25], and a diversity of vesicular components, including elements defined as rhoptries and micronemes, as well as additional membrane-bound structures in some cases [5], [6], [18], [20], [21], [23], [26]–[33]. In these organisms, any association between the apical complex and flagellar apparatus can be examined directly, which is what we describe here. Previously we described *Psammosa pacifica*, a new lineage of predator that branches near the split between apicomplexans and dinoflagellates at the base of the dinoflagellate lineage, and possesses the archetype apical complex with pseudoconoid in the flagellated cell [34]. The apical complex of *P. pacifica* is important not only in the context of the apicomplexans, but also its subsequent early evolution in dinoflagellates. Dinoflagellates lack a structure readily recognizable as the apical complex,[27]–[29], [31], but it has been hypothesized that it was completely lost, or rather drastically changed in morphology during their early evolution. Specifically, some dinoflagellates possess an intracellular structure called peduncle, which is used for myzocytosis [19], and it has been postulated that the peduncle may be homologous to the apical complex [18], [27] based on its function and the presence of the peduncle-associated microtubular basket/strands, which are hypothesized to be homologous to the microtubular apical complex [35]–[39].

To resolve these issues in early apical complex evolution, we used serial TEM tomography for the first time to reconstruct the 3D structure of an apical complex, that of *P. pacifica,* and to directly examine its association with the flagellar apparatus. The resulting 3D model shows: (1) the details of its eight-microtubular pseudoconoid, with additional microtubules running longitudinally towards the flagellar apparatus, (2) two clusters of microneme-like organelles associated to the microtubular pseudoconoid, and (3) an association between the apical complex and a “gullet” positioned at the cell apex. In addition, we used conventional TEM and serial ultrathin sections to confirm that *P. pacifica* possesses a flagellar apparatus comparable with those of dinoflagellates and perkinsids, notably with R1 *sensu* Moestrup 2000 (or longitudinal microtubular root, LMR) connected to the longitudinal basal body, and R4 associated with transverse striated root (TSR) connected to the transverse basal body, and, most importantly, that the apical complex is associated with the microtubule bundle that is connected to R1 via electron dense material. Overall, we demonstrate a connection between the apical complex and the flagellar apparatus, and by providing its exact position within the context of the universal flagellar apparatus system also provide evidence for the homology of the apical complex to the dinoflagellate peduncle.

## Results

### TEM Tomography and 3D Reconstruction of the Apical Complex

**Figure 1:**
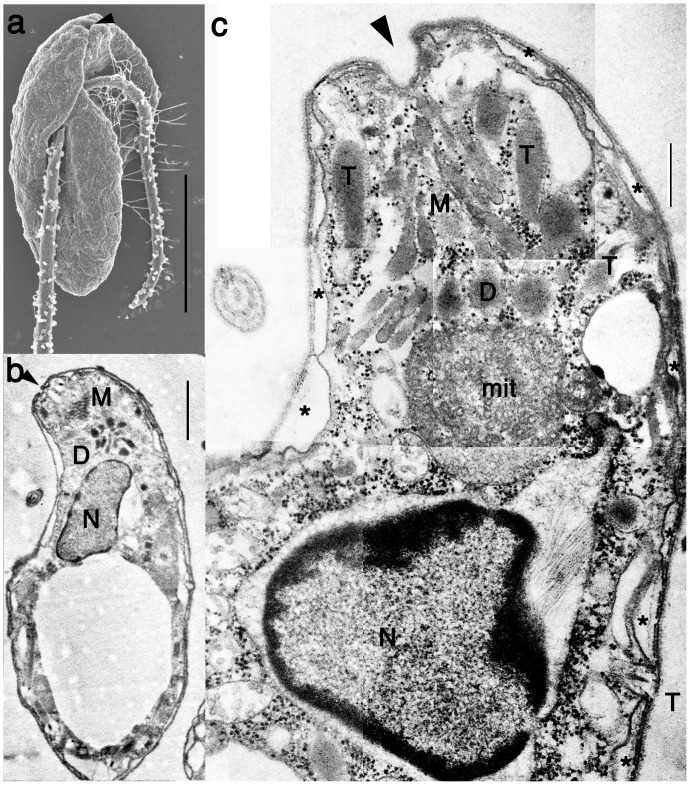
General morphology of *Psammosa pacifica*. **a**. Surface structure of the ventral side of *Psammosa pacifica*, showing two flagella inserted subapical region of the ventral side. The opening of the gullet (arrowhead) is located at the cell apex. **b**. A longitudinal section of the cell showing a cluster of micronemes (M), dense vesicles (d) and the nucleus (N). **c**. The longitudinal section of the apical region shows a cluster of micronemes (M), dense vesicles (D) between the opening of the gullet (arrowhead) and a mitochondrion (mit). Single membrane bound trichocysts (T) are located near the cluster of micronemes. Alveoli vesicles (asterisk) are absent at the gullet and trichocysts. N: nucleus. Scales: 5 µm in **a**, 1 µm in **b**, 500 nm in **c**.

*Psammosa pacifica* is a barley shaped cell with two flagella subapically inserted in the ventral side throughout the life cycle (Fig. 1a). The flagellate cell possesses the apical complex composed of microtubules and electron dense vesicles in the apical area (Fig. 1b–c).

**Figure 2:**
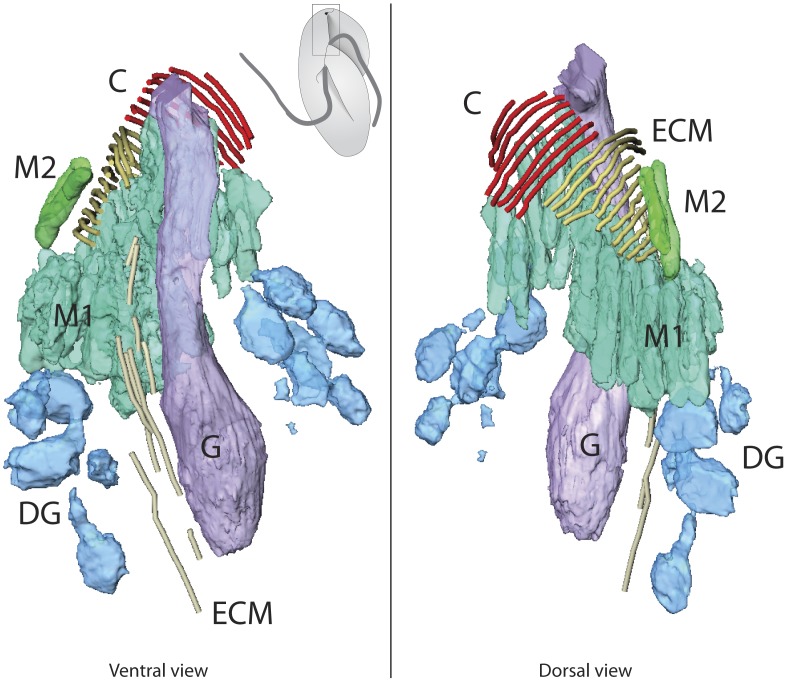
Tomographic reconstruction of the *Psammosa pacifica* apical complex (Cell 1). The microtubule component of the *P. pacifica* apical complex is composed of eight short conoid microtubules (C, red) and a similar number of extended conoid microtubules (ECM, brown) that are longer and extend towards the posterior of the cell. The vesicular components includes two clusters of rhoptries; one of which (M1, turquoise) is on the ventral side of the pseudoconoid, and the other (M2, green) is on the right side of ECM. It also includes the large gullet (G, purple) and several spherical dense granules (D, blue).

**Figure 3:**
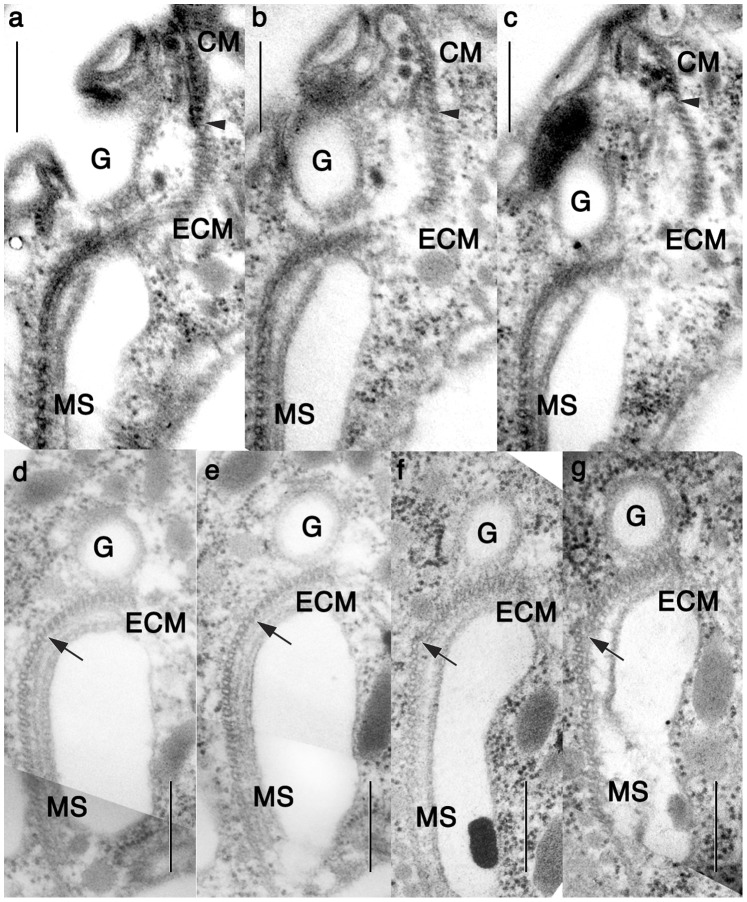
Serial TEM of the extended conoid microtubules (ECM) and microtubular strands. **a**–**g**. Excerpts from a series of serial sections proceeding from the ventral to the dorsal. The extended conoid microtubules (ECM) are aligned between to short conoid microtubules (CM) and microtubule strands (MS), and extend to the posterior towards the basal bodies, following the line of the gullet (G) and a large elongated vesicle. Arrowhead: the boundary between CM and ECM. Arrow: the boundary between ECM and MS. Scale bars = 500 nm.

To further investigate the fine structures and spacial relationships of the apical complex and the flagellar apparatus, we observed two cells by TEM tomography and multiple cells by conventional TEM. Figure 2 shows the reconstructed apical complex overlaid on an illustration of the cell to show its relative position. Movie S1 shows the tomographic reconstruction and 3D model. The apical complex is, not surprisingly, located at the apex of the cell, corresponding to the gullet opening (G) on the lobe that divides the ventral side of the cell to the right-anterior and the left-posterior part. At the anterior end of the pseudoconoid, eight microtubules form a shallow convex sheet open to the ventral side towards the gullet. In addition to the eight conoid microtubules (CM), there is also a band of extended conoid microtubules (ECM: Fig. 2). The conoid microtubules are short and only appear on the dorsal side of the apical complex, and the ECM are immediately posterior and extend further to the posterior of the cell, towards the basal body and the flagellar apparatus. The ECM fall between the gullet and the other long vacuole located on the ventral side of the right anterior part of the cell (Fig. 3). Posterior to the apical complex, the ECM are joined by another set of microtubular strands (MS) that are directly connected to the flagellar apparatus (see below). Between the conoid microtubules and the gullet, lie the micronemes (Fig. 2, 3). Each microneme is clevate, with the narrower anterior neck and the broader, rounded posterior. The micronemes are aligned parallel to each other to form two clusters: one (Mic1) is associated with the inside of the convex curve of the pseudoconoid, and another (Mic2) found posterior to the first cluster. There are no ultrastructurally recognizable difference between the micronemes of Mic1 and Mic2.

**Figure 4:**
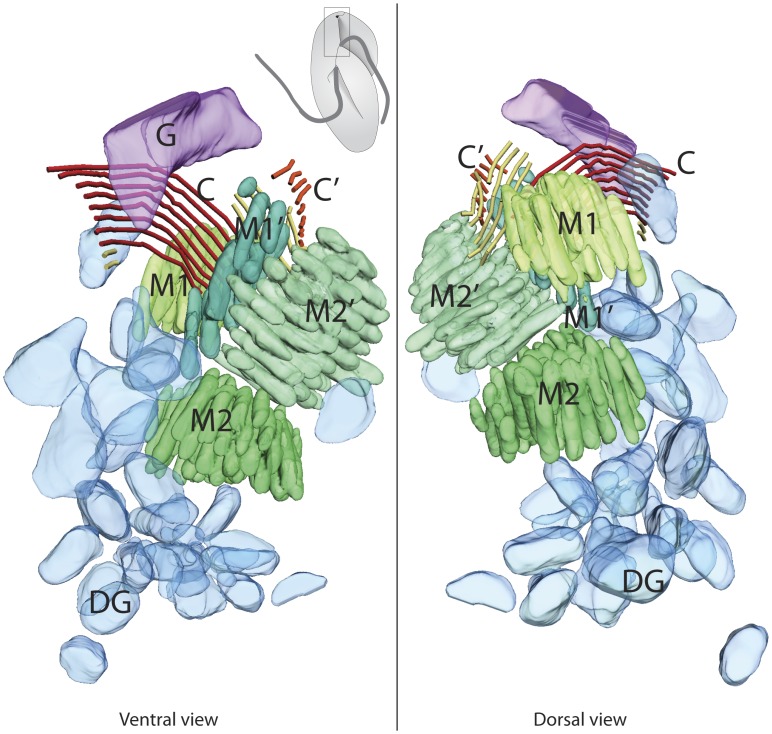
Tomographic reconstruction of mature and nascent apical complexes of *P. Pacifica* (Cell 2). In the second cell where the apical complex was reconstructed by serial tomography, two sets of apical complexes were found, one large and mature, and a second smaller. The structures observed are as in Figure 1, but in each case the number is doubled, so there are two pseudoconoids consisting of eight CMs, two ECMs, four clusters of micronemes, and several spherical dense granules. All abbreviations are as in Figure 1.

Interestingly, the second cell that we observed by serial TEM tomography had a mature apical complex, and also a small nascent apical complex (Fig. 4; Movie S2 for tomographic reconstruction and 3D model). The nascent pseudoconoid (C’) is located left to the mature pseudoconoid (C) and the conoid microtubules of the nascent pseudoconoid are right angle to those of the mature pseudoconoid. There are four clusters of micronemes (Mic1, Mic2, Mic1′, Mic2′) rather than two, altogether consistent with the conclusion that this cell is in the process of duplicating its apical complex, which is normally composed of the pseudoconoid and two clusters of micronemes.

### Flagellar Apparatus

The overall features of the flagellar apparatus of the *P. pacifica* is comparable to that of the dinoflagellates. In this article, we primarily adhere to the universal numbering system of Moestrup 2000, supplemented by the traditional dinoflagellate terminology, as substantial studies have been done before the standardization of the terminology of flagellar apparatus components [38].

**Figure 5:**
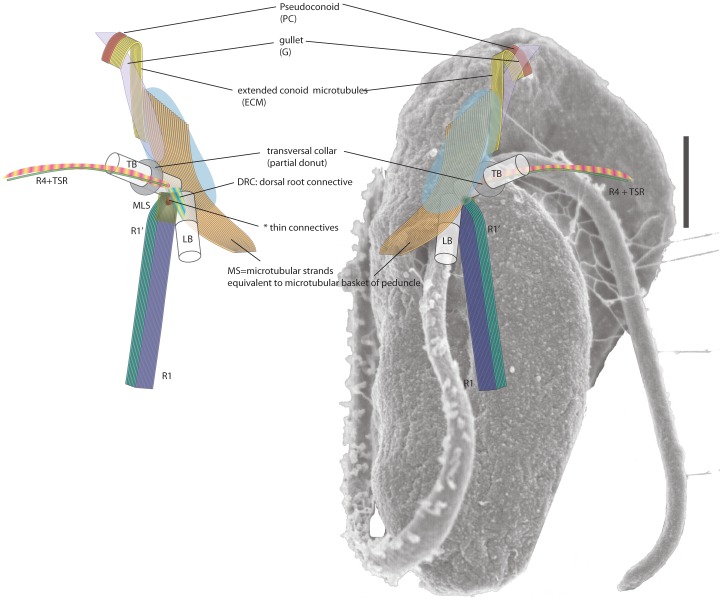
Overview of the apical complex and flagellar apparatus of *P. pacifica*. Dorsal view. **b.** Ventral view. The pseudoconoid (C) consists of eight conoid microtubules and a band of extended conoid microtubules (ECM) that are positioned on the ventral side of the gullet and extend posteriorly towards the basal body. The ECM meet the microtubular strands (MS) that bypass the ventral side of the basal bodies along the ridge of right anterior part of the cell. The MS is connected to the 6 ventral microtubules of the R1/LMR. R1/LMR is lined with a fibrous sheet on the dorsal side and is connected to the longitudinal basal body (LB). A fibrous connective (dorsal root connective: DRC) material on the left side of LB connect to the posterior side of transverse basal body (TB). The LB and TB are at approximately right angles to one another. TB has a fibrous “collar” (transverse basal body collar: TBC) of partial donut shape.

**Figure 6:**
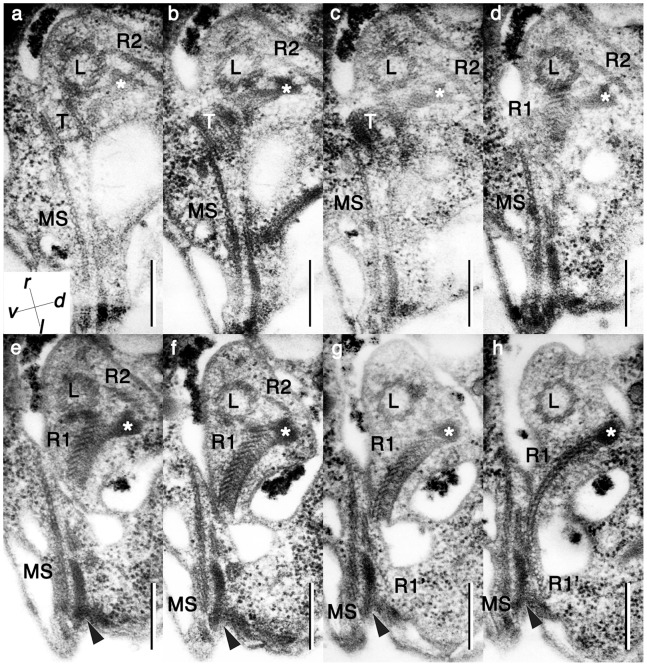
Longitudinal basal body and associated structures. **a**–**h.** series of nearly transversal serial sections, proceeding from anterior to posterior. Arrowheads: dorsal root connective material. Asterisk: thin connective fibers. L: longitudinal basal body. MS: microtubular strands. R1: microtubular root 1, which is also called the longitudinal microtubular root (LMR). R1’: a part of R1 that connects to MS. R2: microtubular root 2, which is composed of 6 very short microtubules. T: transverse basal body. Arrowhead: the fibrous connective between MS and R1. The inset in panel (a) shows the relationship of the plane of these sections to the cell. Scale bars = 500 nm.

**Figure 7:**
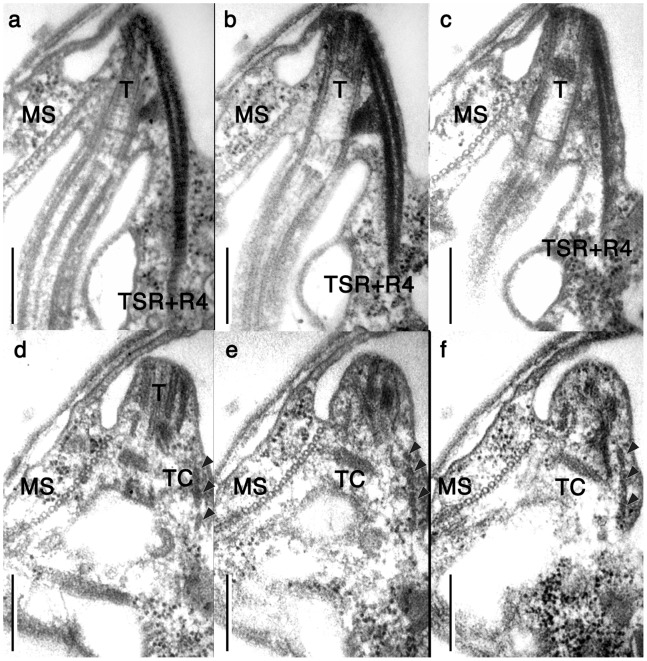
Transversal basal body and associated structures. **a–f.** A series of nearly transversal serial sections, proceeding from anterior to posterior. MS: microtubular strands. R4: microtubular root 4. T: transverse basal body. TC: transverse collar. TSR: transverse striated root. Scale bars = 500 nm.

Figure 5 illustrates the main components of the flagellar apparatus and connections between the pseudoconoid microtubules, the extended conoid microtubules (ECM), and the flagellar apparatus. The transverse and longitudinal basal bodies are inserted sub-apically at nearly right angle to one other on the ventral side of the cell (Fig. 3, 6, 7). Basal bodies are connected by six strands of fibrous connective material on the dorsal side. The transverse basal body (TB) has an electron dense collar near the transition region.

**Figure 8:**
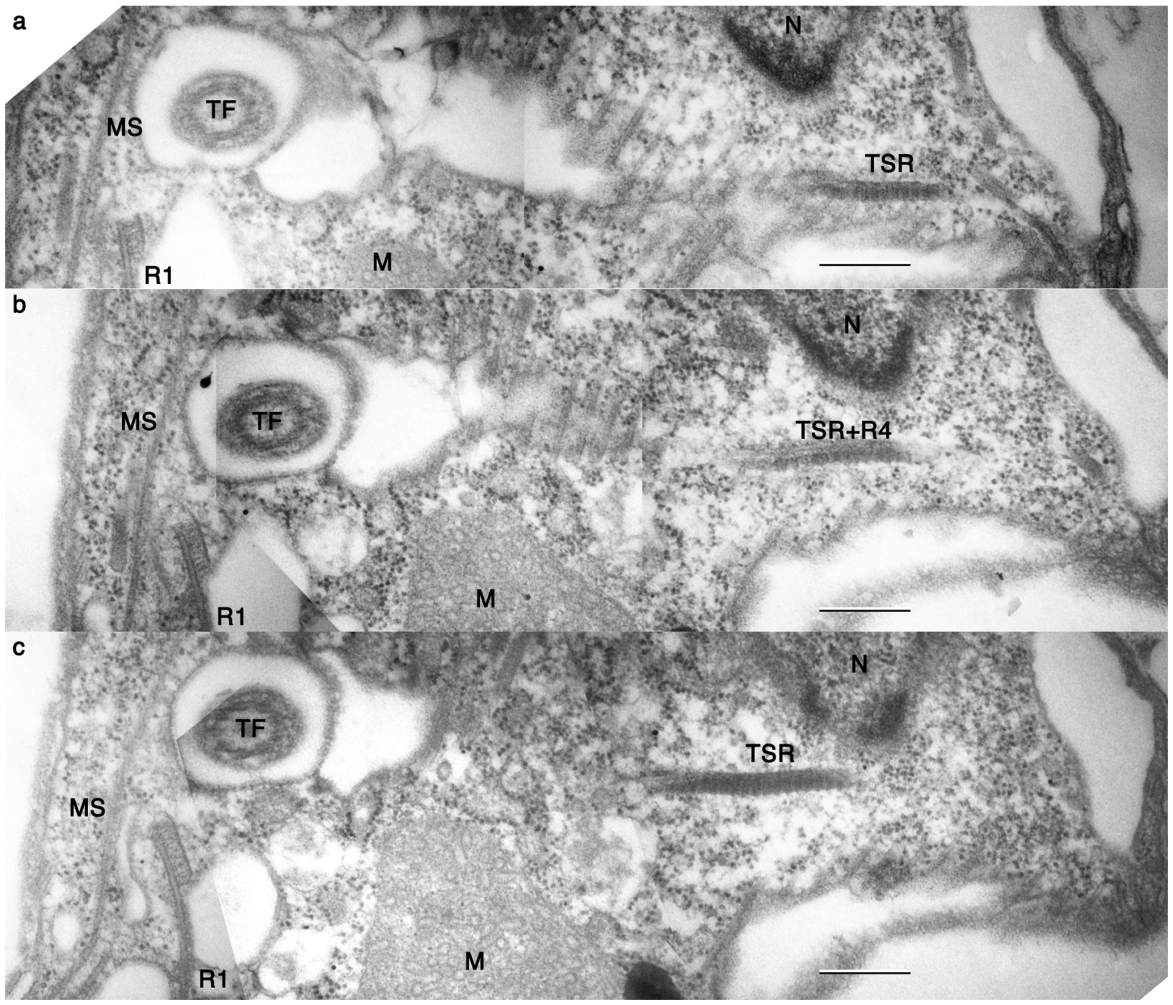
Root 4 and transverse striated root (TSR). **a**–**c.** A series of nearly longitudunal serial sections, priceeding from ventral to dorsal. M: mitochondrion. MS: microtubular strands. N: nucleus. R1: microtubular root 1. R4: microtubular root 4. TF: transverse flagellum. TSR: transverse striated root. Scale bars = 500 nm.

Root 1 (R1) *sensu* Moestrup 2000 is composed of more than 25 microtubules, of which 15 microtubules are connected to the dorsal side of the longitudinal basal body (Fig. 6). The proximal end of R1 is lined with an electron dense sheet. Thin connecting fibers join the electron dense sheet and the transverse basal body. The proximal end of the approximately ten remaining root 1 microtubules (R1’) separate from the longitudinal basal body (LB) and are connected to the wide band of microtubule strands (MS) via electron dense materials at the posterior region of the proximal end of the basal body. The MS bypass the ventral side of the basal body along the ridge of the right anterior part of the body between the dorsally located gullet and the ventrally located vacuole, and join the ECM (Fig. 3). There are six short microtubules on the dorsal side of LB to form root 2 (R2), which are connected to TB, LB and the electron dense sheet of R1 (Fig. 6; arrowheads). Root 3 is missing in *P. pacifica*, and Root 4 (R4) is single microtubule associated with transverse striated root (TSR) (Fig. 3, 7,8). R4 and the TSR are connected to the dorsal side of the transverse basal body by fibrous connective material (Fig. 7). This root extends vertically to cross the left anterior part of the cell (Fig. 8). There is no obvious connection between R4 and surface microtubules that are located beneath the alveoli vesicles (Fig. 3).

## Discussion

### Comparison of the Flagellar Apparatus across Myzozoans

**Figure 9:**
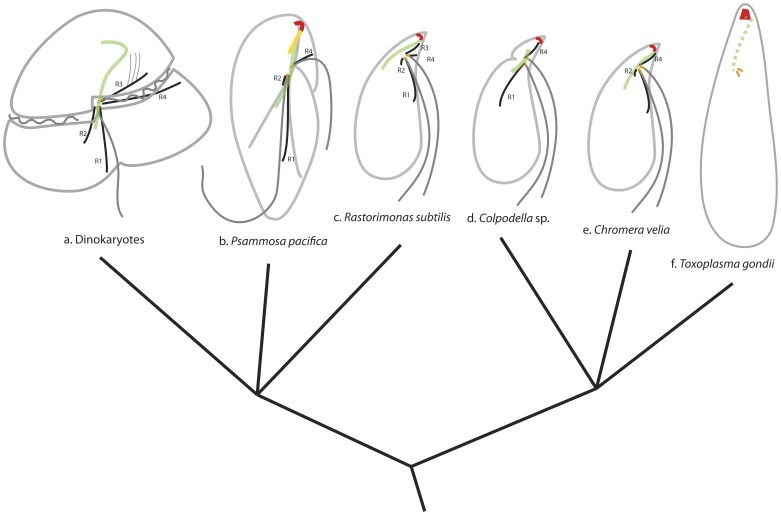
Comparison of the flagellar apparatus and the apical complex in myzozoans. **a.** General dinokaryotes. **b.** *Psammosa pacifica* (this study). **c.** *Rastorimonas subtilis* [21]. **d.** *Colpodella vorax* [20]. **e.** *Chromera velia* [49], **f.** *Toxoplasma gondii* [16]. R1: root 1; R2: root 2; R3: root 3; R4: root 4. Green: bypassing microtubule strands (solid line) or SF-assemblin containing fibre (broken line); red: pseudoconoid or conoid; orange: basal bodies or centorioles.

Figure 9 summarizes the organization of the flagellar apparatus and the apical complex for characterized myzozoans. Among myzozoans, the ultrastructure of the flagellar apparatus of the dinoflagellate is well established (reviewed in [14], [38], [40], [41]). Dinoflagellate cells are comprised of two subregions: the epicone (anterior of the cell) and the hypocone (the posterior). The cell has two flagella inserted on the dorsal side of the junction between the epicone and the hypocone. In dinoflagellates, there are two easily recognizable flagellar roots; root 1 (R1), which is connected to the ventral side of longitudinal basal body, and root 4 (R4), which is accompanied by the transverse striated root (TSR) and is connected to the dorsal side of the transversal basal body. In addition to these two roots, they have a single microtubular root 3 (R3) root that is connected to the ventral side of the transverse basal body. In some species, a single microtubular root 2 (R2) is also present and connected to the dorsal side of the longitudinal basal body. Other notable features are “collars” around basal bodies. In most dinokaryotes, both longitudinal and transverse basal body are accompanied by a striated fibrous structure. Collars and the fibers between them (often striated) contain centrin molecules, and are hypothesized to be involved in the flagellar movement [40].

*Oxyrrhis marina* is one of the basal lineages of dinoflagellates. *O. marina* lacks the microtubular strands/basket, perhaps because *O. marina* does not feed by myzocytosis, but rather by phagocytosis using a bulging structure called “tentacle” located near the flagellar insertion. Here, an additional V-root emerges on the ventral side of the longitudinal basal body and extends into the tentacle, where numerous profiles of electron dense vesicular structure have been observed [42], [43]. Elongated electron dense vesicles were also reported from *Amoebophrya*, a parasitic flagellate that belongs to a basal dinoflagellate lineage, syndinians [44]. A possible association between the microtubular basket and the flagellar apparatus remains unknown in *Amoebophrya*, since the short-lived invasive stage lacks flagella or a complex flagellar apparatus, analogous to the apicomplexans.

The microtubular roots of *P. pacifica* can be viewed as a variation of those of the dinoflagellates: R3 is absent, R2 is shortened, R4 is present with TSR and R1 retains the connection to the pseudoconoid via the extended conoid microtubules (ECM). The extension of R1 and the ECM in *Psammosa* is most likely homologous to the bypassing MS of the dinoflagellate peduncle, based on the spacial relationships to the basal bodies in the cell. In dinoflagellates, the MS is associated with the peduncle, though the orientation of the peduncle in relation to the microtubular strands/basket varies among species; the peduncle emerges anterior to the basal bodies in some cases [36], [45], while in others it emerges posterior [35], [37], [38], [46], [47].

Except for *Perkinsus* and *Psammosa,* dinoflagellates lack a structure readily recognizable as the apical complex. The functional similarity but the weak morphological resemblance between the apical complex and the dinoflagellate peduncle has been noted [18], [25], [27]. However, their homology has been a subject of debate [48]. The demonstration here of a physical connection between the flagellar apparatus and the apical complex in *Psammosa* via R1+ECM strongly supports the homology between the dinoflagellate peduncle and the apical complex. Dinoflagellates, perkinsids, colpodellids and *Psammosa* all have a bundle of microtubules that bypass the dorsal side of basal bodies from the right posterior to the left anterior of the cell.

Among perkinsids, additional microtubules that bypass the basal bodies, split into two strands, and extend anteriorly towards the apical complex have been reported in *Parvilucifera infectans* [27]. However, a connection between these additional microtubules and the apical complex was not reported. In *Perkinsus marinus,* on the other hand, a few microtubules of the pseudoconoid extends posteriorly toward the flagellar apparatus, but again a possible connection of these microtubules to the flagellar apparatus was not reported [30], [31]. Taken together with our results, we suggest the perkinsid apical complex is also physically linked to its flagellar apparatus.

In *Colpodella vorax*, a flagellate thought to be closer to the apicomplexans where the apical complex and flagellar apparatus are found simultaneously [20], two microtubular roots, R1 (“pR”) and R4 (“aR”) are associated with the posterior basal body and the anterior basal body, respectively. In addition, there is a microtubular strand (“oR”) obliquely passing the flagellar apparatus. R4 extends to the anterior apex, beyond the area with subsurface pellicular microtubules, and locate near the open side of the C-shaped pseudoconoid [20].

Interestingly, during the course of our work, Portman et al. reported a similar microtubular extension near the apical complex of *Chromera velia* [49], a photosynthetic relative of apicomplexans. In this report, one view (their figure 4B–E) shows the bypassing microtubules (as opposed to the description in the text, which claims that these microtubules terminate at the anterior basal body) that meet the apical complex at a right angle. These microtubules probably correspond to the previous observation of a “double layered pseudoconoid” by Oborník et al (Fig. 36, 38 in [50]). It is unclear if the posterior end of the bypassing microtubules (unannotated in their figure 4B) has any connection to R1, as we show in *Psammosa* and is known in dinoflagellates. They also show R4 extending from the dorsal side of the anterior flagellum, which was originally interpreted to show that “part of the subpellicular microtubule array lies alongside the anterior flagellar groove”. R4 is shown extending near the pseudoconoid, analogous to *C. vorax* [20]. R2 is similar to that of *Psammosa* in that it consists of short microtubules that lie between the posterior and the anterior basal bodies.

### Replication of the Apical Complex and Possible Presence of the Apical MTOC in *Psammosa*

The second *Psammosa* cell that we observed by tomography showed the duplicated clusters of micronemes and the nascent pseudoconoid. The principle components of the two apical complexes are the same; the eight-microtubular pseudoconoid and two clusters of micronemes. The nascent apical complex is not connected to the ECM, which suggests the duplication of the pseudoconoid microtubules are formed at the apex. In the case of *T. gondii*, an independent ring-shaped MTOC for subpelicullar microtubules, named the apical polar ring (APR) exists at the anterior of the apical complex [51], [52], [53]. It would be interesting to know if *Psammosa* has a similar apical MTOC for pseudoconoid formation. *Psammosa* cells replicate by horizontal binary division [34], identical to that of *Oxyrrhis marinus*, which has a subsurface ring MTOC area on the division plane [54]. In addition, *Psammosa* must have an MTOC for the basal body and flagellar root duplication.

### The Origin and Evolution of the Apical Complex

Emerging evidence based not only on the morphology but also the molecular composition suggests the apical complex and the flagellar apparatus are connected. Francia et al [16] recently reported that a non-microtubular, fibrous connection between the apical complex of *T. gondii* and the MTOC contains homologues of SF-assemblin, one of the components of flagellar apparatus found in green algae/plants and a stramenopile protists [55], [56]. In green algae, SF-assemblin is localized at the striated microtubule-associated fiber (SMAF) associated with the microtubular flagellar roots [57], [58]. In *T. gondii*, the fibrous structure that contains SF-assemblin homologues does not show striated pattern, but still maintains the connection between the inner microtubular pair of the apical complex and the centrioles, and therefore ensures the even inheritance of the organelles to daughter cells during division. In *Psammosa*, the apical complex is directly connected to the longitudinal basal body through series of connected microtubular structures; ECM, MS, and R1, which are connected to one another via fibrous connections, none of which is striated. In *Colpodella vorax* and *Chromera velia,* R4 is close to the apical complex, though not directly connected. R4 in *Psammosa*, dinoflagellates, and perkinsids has a striated fibre, though in *Colpodella* and *Chromera*, the associated fibre does not have striation. It would be of interest to know if R1 or R4 associated fibers contain SF-assemblin.

The SAS-6 like protein (SAS6L) [17] is another interesting candidate for the apical complex-flagellar apparatus connection. SAS6L is localized at the preconoidal ring of *T. gondii* tachyzoites, whereas located at the basal plate of the flagella in *Trypanosoma brucei*. The exact function of SAS6L remains enigmatic and is not essential in tachyzoites. If it is involved in basal body formation, as is suggested in *Tr. brucei*, SAS6L might have a primary role in the other stages of *T. gondii* such as in microgametes flagellar formation. The location of any SAS6L homologues in *Psammosa* would also be interesting in the context of MTOC and microtubular populations.

**Figure 10:**
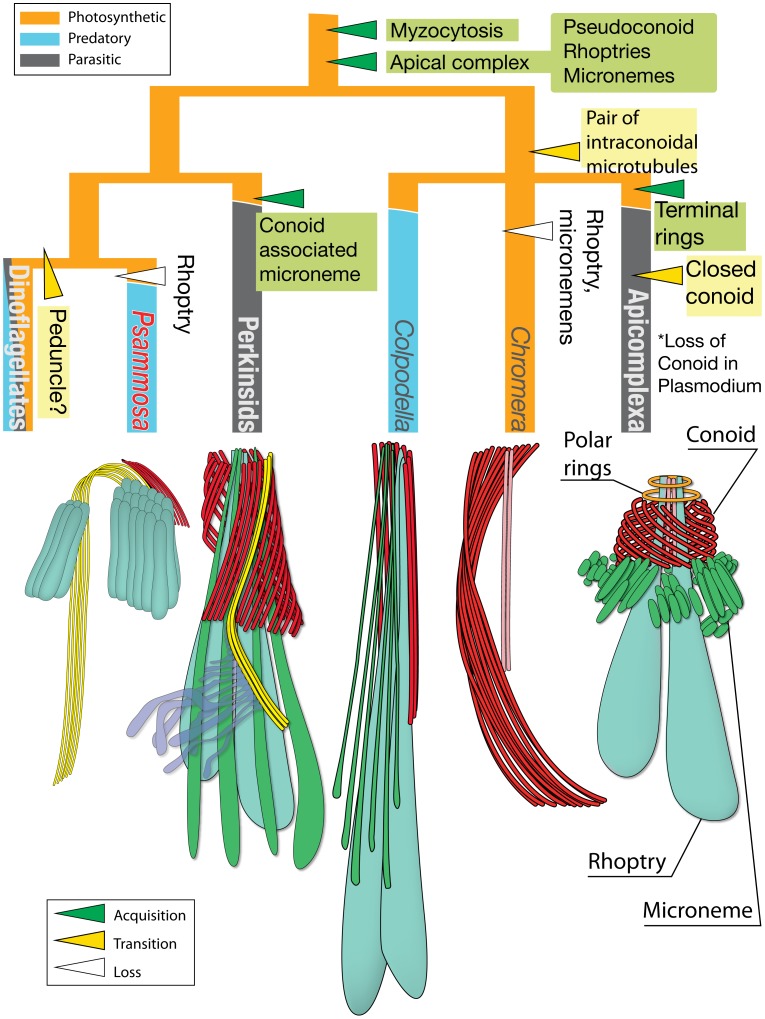
Character evolution of the apical complex among myzozoans. Color of the tree branches indicate trophic strategy of the linages; orange: photysynthetic, blue: predatory; grey: parasitic. Character evolution is indicated with triangles; green: acquisition of a character, yellow: transition of a character, white: loss of a character.

Figure 10 shows a reconstruction of character evolution of the apical complex. The apical complex traces back to the common ancestor of the apicomplexan parasites and the dinoflagellates, i.e. an ancestral myzozoan. The “archetype” apical complex is composed of a pseudoconoid, or open-sided conoid [25], which are still found among colpodellids, perkinsids, and *Psammosa*. In the apicomplexan parasites, the conoid developed the truncated conical shape with a pair of intra-conoidal microtubules seen today, and the polar ring was added to the anterior end. *Chromera velia* also is an interesting intermediate variant, in that the pseudoconoid is open-sided, but with a pair of intra-conoidal microtubules and the additional layer of bypassing microtubules [49], [50]. It is unclear if either layer of the microtubules are connected to the other cytoskeletal elements in *C. veila*, and further study is needed. In the dinoflagellates, the structure is most likely developed to peduncle as discussed above.

The majority of apical complexes examined so far have both rhoptries and micronemes, and, in perkinsids, also conoid associated micronemes (CAM). However, in several cases one or more of these structures is absent, so losing vesicular components of the apical complex appears to be common. For example, *Haemogregarina magna* retains only micronemes [59], *Cryptosporidium* sp. ex *Ruditapes decussatus* retains only rhoptries [60], and *Colpodella pugnax* retains only rhoptries [23]. These are scattered across the different lineages of myzozoans, suggesting several independent losses. Indeed, the apicomplexan *Theileria parva* changes its vesicular components depending on life stage-specific host invasion strategies; sporozoites and merozoites lack micronemes, whereas ookinetes retain both rhoptries and micronemes (for review, [61]). In the apicomplexans, rhoptry proteins are injected into the host cell, whereas microneme proteins are secreted on the surface of the parasite itself to mediate the host-parasite interaction [62]. *Psammosa* possesses a single type of elongated, rhomboid shaped vesicle, which we referred to as micronemes as our preliminary investigation of *Psammosa* EST includes genes with conserved domains that are found in microneme proteins in various apicomplexans (unpublished data). As a predator, *P. pacifica* does not require the same interactions with its prey, so this may have led to a reduction of its vesicular complexity. Conversely, however, the apical complex of *P. pacifica* is unique in that it is associated with the gullet, a permanent invagination at the apex of the cell, and a large vacuole. In *Chromera*, a large vacuole, which is a part of the endomembrane system situated posterior to the pseudoconoid, though without an opening as found in *Psammosa* [49].

### Myzocytosis and the Origin of Parasitism

The function of the apical complex is perceived primarily as invasion, but myzozoans include lineages of diverse trophic strategies: parasitism, predation, photoautotrophy, and mixotrophy. Not only parasites, but also free-living grazers and photosynthetic algae retain the apical complex, obviously for a variety of functions. In predatory and mixotrophic lineages, the apical complex is used for myzocytotic feeding. In *C. velia*, a photosynthetic relative of the apicomplexans that is thought to be a coral endosymbiont [63], the apical complex may have a role in establishment of endosymbiosis [64]. The plastid of dinoflagellates and apicomplexans clearly preceded these lingeages [65], suggesting the ancestral myzozoan was most likely either an endosymbiont or a mixotroph, so parasitism based on the apical complex probably evolved much later.

### Concluding Remarks

We reconstructed the three dimensional structure of the apical complex and flagellar apparatus of the basal sister to dinoflagellates, *P. pacifica*. This provides the first demonstration of a direct microtubular link between these two cytoskeletal structures. Based on an ultrastructural comparison of the flagellar apparatus and associated structures across a wide range of alveolates, we also conclude that the peduncle, a feeding apparatus found in myzocytotic dinokaryotes, is probably homologous to the apical complex. Further understanding of the apical complex of *P. pacifica* at the molecular level, as well as the investigation of the 3D structure of the apical complex in related lineages and molecular evidence for proteins shared between the apical complex and the flagellar apparatus will all elucidate the relationship between and evolution of these characters in both dinoflagellates and apicomplexans, as well as the emergence of virulence in these lineages.

## Materials and Methods

### Cell Culture

Cells of *Psammosa pacifica* were cultured in K+CoQ medium at 17°C with the presence of *Spumera* sp. as the prey [34].

### Sample Preparation for TEM and TEM Tomography

Serial ultrathin and thin section TEM was performed on actively growing *Psammosa pacifica* Okamoto et al 2012 culture that was semi-simultaniously fixed with 2.5% Gutarardehyde and with 0.01% Osmium tetroxide in sea water (final concentration, respectively). Cells were then rinsed once with distilled water, dehydrated through ethanol series, then embedded in SciPon resin. Serial ultrathin sections (50 nm thickness) and serial thin sections (200 nm thickness) were collected on Formvar-coated slot grids.

### TEM Observation

Ultrathin sections were observed under a Hitachi H7600 electron microscope (Hitachi, Japan). Contrast was adjusted within Adobe Photoshop CS5 software (San Jose, CA).

### TEM Tomography and 3D Modeling

Ribbons of serial thin sections (200 nm thick) were collected on formvar coated slot grids, then post-stained with aqueous uranyl acetate and Renyold’s lead citrate. Colloidal gold particles were deposited on both surfaces of the sections for use as fiducial markers during subsequent image alignment. Sections were viewed in an FEI Tecnai-G2 electron microscope operating at 200 KeV, and images recorded digitally with a FEI Eagle 4K bottom mount CCD camera (FEI Tecnai, OR) using a pixel size of 1.2 nm. Tilt series were recorded with automated methods for image montaging, data acquisition and image alignment as the sample was serially tilted along single axis from at −65° to +65°, by 1° angular increments over a range between +40° and −40°, and by 2° angular increments over the rest. 3D distributions of stain density (tomograms) were calculated from each tilt series on FEI Xpress3D reconstruction (FEI Tecnai, OR). Tomographic reconstructions were aligned with each other and combined to produce a single dual-axis 3D reconstruction on Amira v.5.4.0 software. Tomograms from adjacent sections were aligned to each other, then subcellular structures and membranes within the 3D volumes were analyzed and modeled.

## Supporting Information

Movie S1 **Tomographic reconstruction and 3D model of the apical complex of** ***P. pacifica*** **- Cell 1.**
(MP4)

Movie S2 **Tomographic reconstruction and 3D model of the apical complex of** ***P. pacifica*** **- Cell 2.**
(MP4)
